# A single-centre retrospective analysis of cinacalcet therapy in primary hyperparathyroidism

**DOI:** 10.1530/EC-21-0258

**Published:** 2021-10-14

**Authors:** Daniel Bell, Julia Hale, Cara Go, Ben G Challis, Tilak Das, Brian Fish, Ruth T Casey

**Affiliations:** 1 Department of Pharmacy, Cambridge University Hospital NHS Foundation Trust, Cambridge, UK; 2 Department of Endocrinology, Cambridge University Hospital NHS Foundation Trust, Cambridge, UK; 3 Department of Radiology, Cambridge University Hospital NHS Foundation Trust, Cambridge, UK; 4 Department of Head and Neck Surgery, Cambridge University NHS Foundation Trust, Cambridge, UK; 5 Department of Medical Genetics, Cambridge University, Cambridge, UK

**Keywords:** cinacalcet, calcium, primary hyperparathyroidism

## Abstract

Primary hyperparathyroidism (pHPT) is a common endocrine disorder that can be cured by parathyroidectomy; patients unsuitable for surgery can be treated with cinacalcet. Availability of surgery may be reduced during COVID-19, and cinacalcet can be used as bridging therapy. In this single-centre retrospective analysis, we investigated the utility and safety of cinacalcet in patients with pHPT receiving cinacalcet between March 2019 and July 2020, including pre-parathyroidectomy bridging. We reviewed and summarised the published literature. Cinacalcet dosages were adjusted by endocrinologists to achieve target calcium < 2.70 mmol/L. Eighty-six patients were identified, with the most achieving target calcium (79.1%) with a mean dose of 39.4 mg/day (±17.1 mg/day) for a median duration of 35 weeks (1–178 weeks). Calcium was normalised in a median time of 5 weeks. The majority of patients commenced cinacalcet of 30 mg/day (78 patients) with the remainder at 60 mg/day (8 patients). Forty-seven patients commencing lower dose cinacalcet (30 mg/day) achieved target calcium without requiring 60 mg/day. Baseline PTH was significantly higher in patients requiring higher doses of cinacalcet. 18.6% of patients reported adverse reactions and 4.7% discontinued cinacalcet. Patients treated with cinacalcet pre-parathyroidectomy required a higher dose and fewer achieved target calcium compared to medical treatment with cinacalcet alone. Post-operative calcium was similar to patients who were not given pre-parathyroidectomy cinacalcet. In summary, cinacalcet at an initial dose of 30 mg/day is safe and useful for achieving target calcium in patients with symptomatic or severe hypercalcaemia in pHPT, including those treated for pre-parathyroidectomy. We propose a PTH threshold of >30 pmol/L to initiate at a higher dose of 60 mg/day.

## Introduction

Primary hyperparathyroidism (pHPT) is a common endocrine disorder with a prevalence of 1–4 per 1000 and a 3 to 1 female to male ratio (1). It is characterised by inappropriate and excessive secretion of parathyroid hormone (PTH) from one or more parathyroid glands resulting in hypercalcaemia. Chronic hypercalcaemia is often asymptomatic but can lead to classical renal and skeletal complications, as well as neurocognitive and cardiovascular effects (2).

The only curative treatment option for pHPT is parathyroid surgery (PTX), which is indicated according to international guidelines in those with symptomatic hypercalcaemia, renal/skeletal complications or a diagnosis of pHPT in patients less than 50 years (2). Some patients meeting criteria for surgery do not proceed to PTX due to increased surgical and/or anaesthetic risk, or patient preference. In this cohort of patients, medical management with cinacalcet can be considered, and cinacalcet therapy is routinely commissioned in these patients in England (3).

Cinacalcet is a positive allosteric modulator of the calcium-sensing receptor (CaSR) on the chief cell of the parathyroid gland. It increases the sensitivity of the CaSR to extracellular calcium which directly reduces the secretion of PTH and indirectly reduces serum calcium. While a variety of studies have demonstrated that cinacalcet is effective in reducing serum calcium, improvement in the clinical sequelae of pHPT has not been consistently observed (4, 5). Accordingly, cinacalcet is used to alleviate symptoms of hypercalcaemia and avoid ensuing hospitalisation in symptomatic patients, or those with severe hypercalcaemia (defined as >3.00 mmol/L by NHS England (6)). It is licensed at an initial dose of 60 mg/day, although the largest prospective study to date showed effectiveness with a mean dose <60 mg/day (PRIMARA study) with adverse drug reactions resulting to discontinuation in 7.6% of patients (7).

Cinacalcet has also been studied in the pre-PTX setting to normalise hypercalcaemia or help in the symptomatic management of patients prior to surgery (8, 9). It is recognised that the ongoing 2019 novel coronavirus (COVID-19) pandemic will reduce the availability of elective PTX, and guidance from the European Society for Endocrinology supports using cinacalcet off-label as a bridge in selected patients awaiting PTX (10).

At our centre, cinacalcet for pHPT is managed on a shared care basis following the pathway set out in the Supplementary Appendix (see section on supplementary materials given at the end of this article) (11), initiated at a dose of 30 mg/day and titrated to achieve a target serum calcium < 2.70 mmol/L by endocrinologists in the pHPT clinic, with long-term prescribing by general practitioners once biochemically stable. In this evaluation, we retrospectively reviewed the utility and safety of cinacalcet in the treatment of pHPT, including its use in patients prior to PTX delayed by COVID-19 and compared our results to those presented in the literature.

## Materials and methods

### Patient selection

All patients seen in the pHPT clinic between March 2019 and July 2020 were assessed for eligibility. Every patient who received a prescription of cinacalcet under the pHPT clinic was taken forward for review, including those who started cinacalcet prior to March 2019 and those who subsequently ceased therapy for any reason.

Eligibility criteria for starting cinacalcet at the pHPT clinic are described in the local shared care pathway summarised in the Supplementary Appendix. Additionally, during the first wave of the COVID-19 pandemic, a small number of patients were treated with cinacalcet who were symptomatic at a lower serum calcium threshold, to avoid hospitalisation.

This study was approved as a service evaluation in Cambridge University Hospitals NHS Foundation Trust (local project ID 9049).

### Data collection

Electronic patient records (EPRs) were accessed in the local e-hospital system to assess the start date of cinacalcet therapy, biochemistry results, date of dose adjustment, adverse effects and date of cessation. Symptoms at presentation and during treatment were not recorded on a specific pro forma in the EPR. Prescriptions for cinacalcet were not directly recorded in the EPR so the date of initiation, adjustment and cessation were taken from EPR notes describing these events.

Biochemistry was assayed in the Trust laboratory and accessed from the EPR: serum calcium (Ca, mmol/L), adjusted serum calcium (aCa, mmol/L), serum phosphate (PO, mmol/L), parathyroid hormone (PTH, pmol/L) and estimated glomerular filtration rate (eGFR, mL/min/1.73 m^2^, capped at 90 mL/min/1.73 m^2^ by the Trust laboratory). External results supplement those from the Trust laboratory in a small number of patients where biochemistry was assayed by external laboratories. The baseline bloods were determined as the last results reported before cinacalcet initiation, and the post-treatment bloods were determined as either the most recent bloods where treatment continues or the final bloods before treatment cessation where therapy ceased for any reason.

Patients were further subdivided for analysis into those receiving lower dose cinacalcet therapy (cinacalcet dose ≤ 30 mg/day (15 mg/day or 30 mg/day)) and those receiving higher dose cinacalcet therapy (cinacalcet dose > 30 mg/day (45, 60 or 90 mg/day]). The data that support the findings of this study are available on request from the corresponding author.

### Statistical analysis

Statistical analysis was performed using free online software (www.socscistatistics.com); a non-paired *t*-test was used in the analysis of post-treatment biochemistry in low vs high dose cinacalcet therapy, and a paired *t*-test was used for the analysis of pre- vs post-treatment biochemistry. A *P* -value of <0.05 was considered statistically significant. Mean values are presented ± s.d. Median values are presented with the range in parentheses.

### Literature search

The Medline database was searched in May 2020 using the following strategy: (cinacalcet or mimpara or sensipar) AND (primary hyperparathyroidism).

## Results

### Cohort characteristics

Three hundrerd and six patients were seen in the pHPT clinic during this period; 86 were started on cinacalcet and taken forward for review. The 220 patients who did not commence cinacalcet either did not meet the inclusion criteria as set out in our shared care policy (see Supplementary Appendix) or elected not to receive medical treatment with cinacalcet; eligible patients were offered PTX. The majority of our cohort was female (72 patients) with a median age of 77 years (31–94 years). Most patients were symptomatic with hypercalcaemia at presentation (64 patients), most commonly muscle pain or weakness (18 patients), bone pain (17 patients), fatigue or lethargy (15 patients), constipation (12 patients), confusion (12 patients) and polyuria (10 patients).

Baseline mean Ca (2.92 mmol/L ± 0.12 mmol/L), aCa (3.01 mmol/L ± 0.11 mmol/L) and PTH (19.5 pmol/L ± 10.12 pmol/L) exceeded the reference range; mean PO (0.88 mmol/L ± 0.18 mmol/L) was in the lower end of the normal range and mean eGFR was normal (71.1 mL/min/1.73 m^2^ ± 18.6 mL/min/1.73 m^2^) (Table 1). Forty-two patients had a baseline Ca or aCa > 3.00 mmol/L. Thirty-seven patients had a baseline Ca or aCa between 2.90 and 3.00 mmol/L. During the first wave of the pandemic, seven patients were treated with a baseline Ca and aCa above the normal range but <2.90 mmol/L in an effort to reduce or avoid hospital attendance. Six out of these seven patients had documented symptoms of hypercalcaemia (confusion in two patients, polyuria/polydipsia in two patients, unspecified in two patients), and the seventh patient had been receiving cinacalcet under a private endocrinologist pre-dating enrolment with the pHPT clinic. However mean baseline chemistry was near identical prior to first wave of the pandemic and within the opening 4 months of the pandemic. The majority of patients commenced cinacalcet 30 mg/day (78 patients) with the remainder at 60 mg/day (eight patients).

**Table 1:** Baseline demographics and symptoms, with baseline biochemistry stratified according to final daily dose of cinacalcet.

Median age (range)	77 (31–94)	
Sex	72 females, 14 males	
Biochemical parameter (units) *Reference range*	Final daily dose of cinacalcet	Baseline mean biochemistry (±s.d.)
Ca *2.08–2.65 mmol/L*	All	2.92 (±0.12)
	≤30 mg	2.89 (±0.15)
	>30 mg	2.95 (±0.13)
aCa *2.20–2.60 mmol/L*	All	3.01 (±0.11)
	≤30 mg	2.99 (±0.13)
	>30 mg	3.06 (±0.13)
PO *0.80–1.50 mmol/L*	All	0.88 (±0.18)
	≤30 mg	0.90 (±0.19)
	>30 mg	0.84 (±0.16)
PTH *1.5–7.6 mmol/L*	All	19.5 (±10.1)
	≤30 mg	16.6 (±6.8)^a^
	>30 mg	27.5 (±13.3)^a^
eGFR^b^ *>60 mL/min/1.73 m2*	All	71.7 (±18.6)
	≤30 mg	69.7 (±17.7)
	>30 mg	77.0 (±20.4)

^a^Indicates statistically significant difference in baseline biochemistry between patients taking ≤30 mg/day cinacalcet and patients taking >30 mg/day cinacalcet; ^b^eGFR results capped at 90 mL/min/1.73 m^2^ by the Trust laboratory.

Those patients requiring higher doses of cinacalcet (>30 mg/day) had significantly higher baseline PTH than those using lower doses (≤30 mg/day; *P* < 0.05). Baseline Ca and aCa were higher, and PO was lower in patients requiring higher doses of cinacalcet (>30 mg/day) but did not reach statistical significance (*P* > 0.05 in all parameters; Table 1).

### Outcomes from cinacalcet therapy

Cinacalcet dosage was adjusted by endocrinologists to achieve a target Ca < 2.70 mmol/L. Overall, 68 patients achieved a target Ca with cinacalcet at a mean daily dose of 39.4 mg ± 17.1 mg/day; no patient received a dose > 90 mg/day (range, 15–90 mg/day). The majority of patients (47 patients) commenced on lower dose cinacalcet (30 mg/day) achieved a target Ca without requiring a full licensed dose of 60 mg/day. The median duration of treatment was 35 weeks (1–178 weeks). In those patients achieving a target Ca, the median time to achieve a target Ca was 5 weeks (1–80 weeks); patients taking lower doses of cinacalcet ≤30 mg/day achieved a target Ca sooner than those taking higher doses of cinacalcet > 30 mg/day, but this was not significant (median 5 weeks (range 1–80 weeks) vs 7 weeks (range 1–47 weeks), *P* = 0.158).

**Figure 1:**
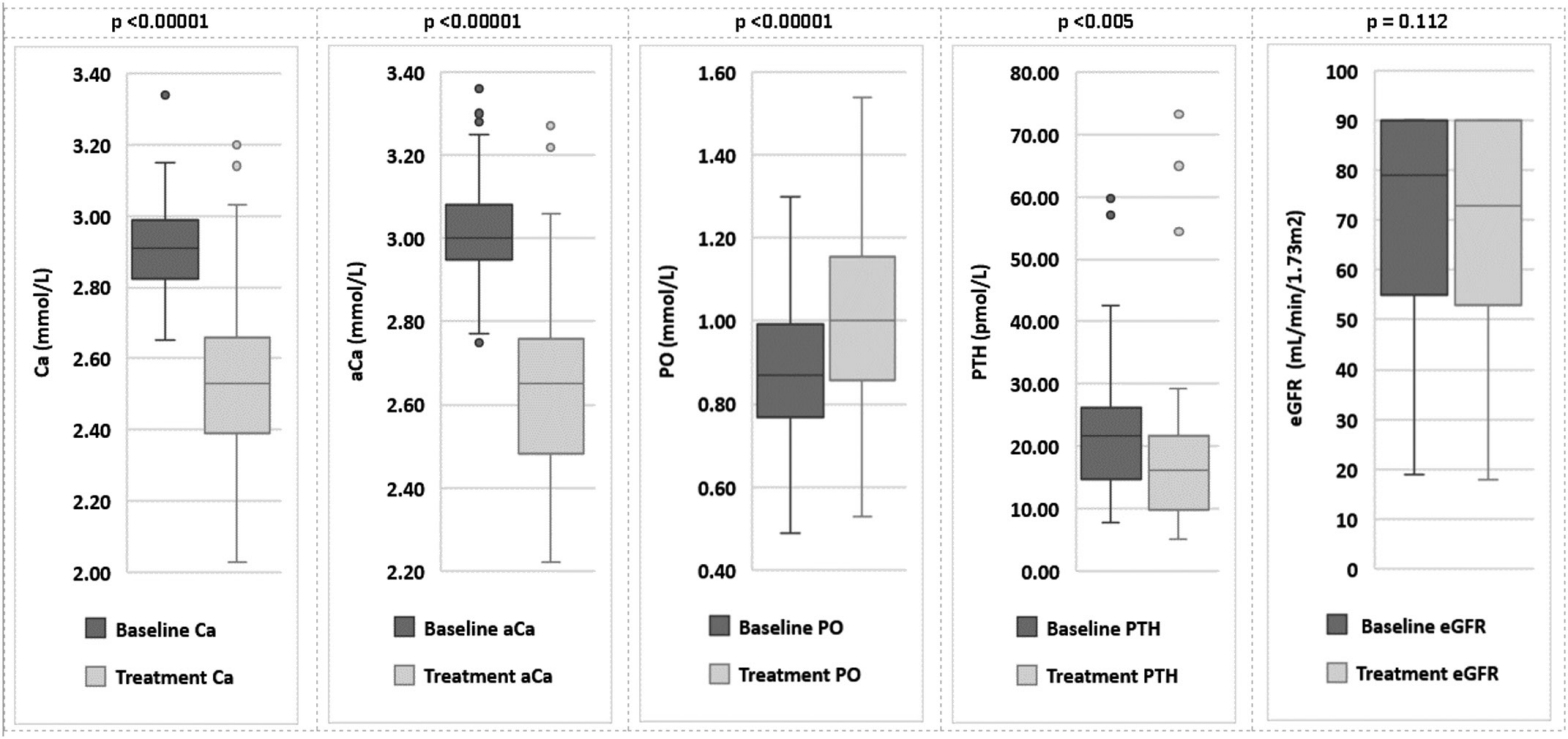
Biochemical responses across the entire cohort, pre- and post-treatment with cinacalcet.

Throughout the treatment period across the entire cohort, mean Ca fell significantly into the normal range (2.52 mmol/L ± 0.22 mmol/L, *P* < 0.001), mean aCa and PTH fell significantly but remained above the normal range (2.63 mmol/L ± 0.21 mmol/L *P* < 0.001 and 18.9 pmol/L ± 13.9 pmol/L, *P* < 0.001, respectively), mean PO rose significantly though remained in the normal range (1.01 mmol/L ± 0.12 mmol/L, *P* < 0.005) and mean eGFR fell but non-significantly (69.7 mL/min/1.73 m^2^ ± 19.8 mL/min/1.73 m^2^, *P* = 0.1123) (Fig. 1). Changes in symptoms associated with hypercalcaemia were not recorded in the EPR such that data could be collected to assess change in symptoms while on cinacalcet.

**Figure 2:**
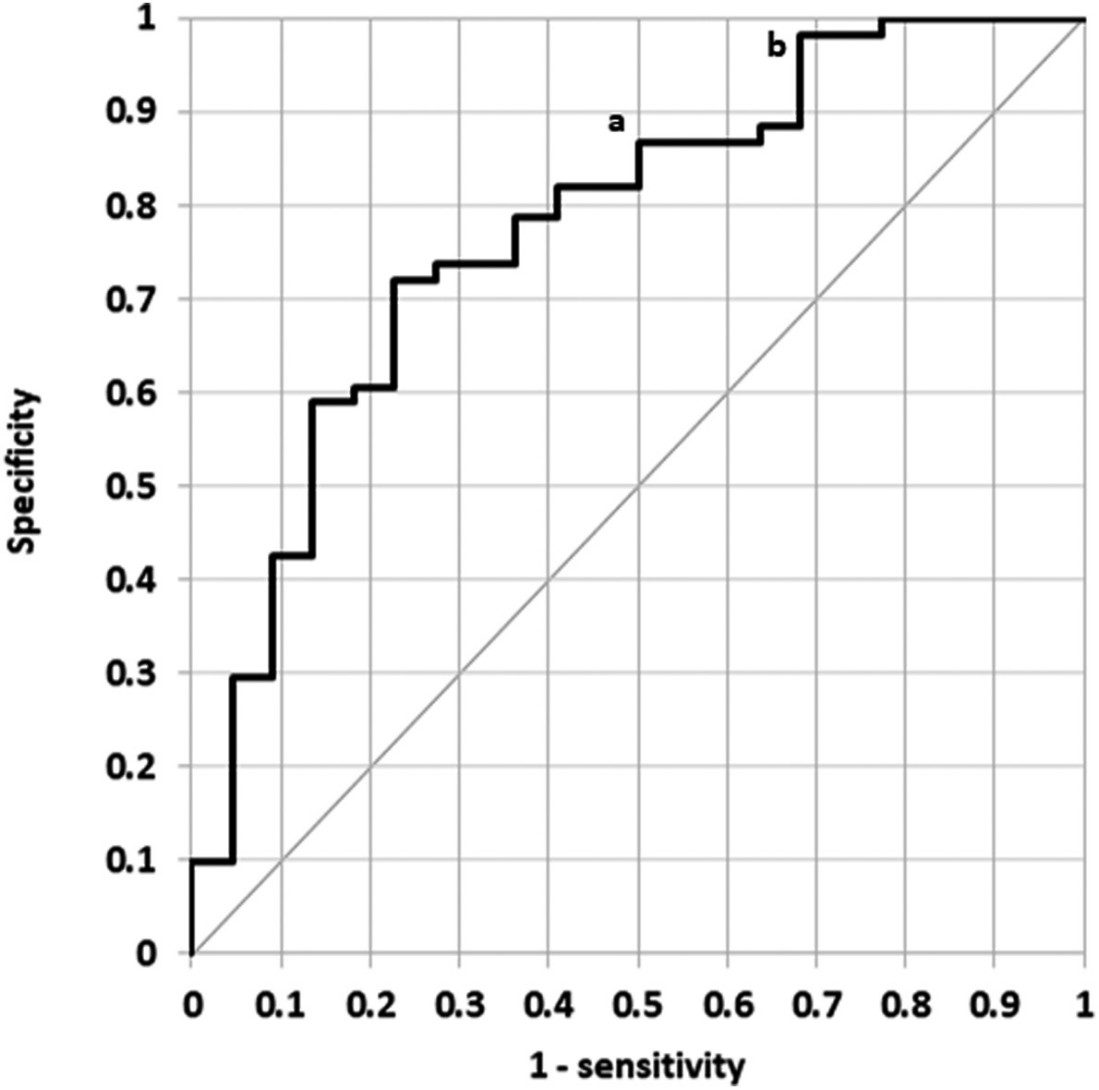
ROC curve; patients requiring cinacalcet > 30 mg/day were considered positives. AUC = 0.777. ^a^PTH 25 mmol/L and ^b^PTH 30 mmol/L.

Additionally, a ROC curve analysis was performed to determine the optimal baseline PTH threshold for initiating cinacalcet at the higher dose of 60 mg/day; positive patients in this analysis were those patients requiring dose adjustment to above initial lower dose of 30 mg/day. A baseline PTH threshold of 25 pmol/L yields 87% specificity and 50% sensitivity, and a threshold of 30 pmol/L yields 98% specificity and 32% sensitivity for indicating those patients likely to require a dose of cinacalcet 60 mg/day (Fig. 2).

Twenty-six patients failed to achieve target Ca on their initial cinacalcet dose, and poor adherence was identified in ten of these patients, most commonly intentional dose reductions/omissions due to nausea (four patients) or headaches (one case), along with problems obtaining a supply of medication (three patients) and unintentional non-adherence due to confusion (two patients).

### Cinacalcet used pre-PTX

Twenty-four patients received cinacalcet prior to PTX (Table 2). Seventeen patients were symptomatic (most commonly fatigue/lethargy in 11 patients, bone pain in 9 patients, polyuria in 4 patients), including 6 patients with Ca and aCa < 3.00 mmol/L. Twenty-one patients were treated for parathyroid adenoma, with one case of parathyroid hyperplasia (single gland removed), one case of ectopic parathyroid adenoma (two previous surgical interventions) and one case of equivocal parathyroid adenoma/hyperplasia. All patients started cinacalcet at a low dose of 30 mg/day, though final doses of cinacalcet prior to surgery were higher in this subgroup than the overall cohort mean (44.3 mg/day ± 21.4 mg/day vs 39.4 mg/day ±17.1 mg/day). Fifteen patients achieved target calcium (mean 2.66 mmol/L ± 0.20 mmol/L, median time to target 6 weeks (4–35 weeks)).

**Table 2:** Summary of 24 patients treated with cinacalcet pre-PTX.

Baseline				Post-treatment with cinacalcet						Post-operative outcomes	
Ca, mmol/L	aCa, mmol/L	PTH, pmol/L	Cinacalcet dose, mg	Change in symptoms	Ca, mmol/L	aCa, mmol/L	PTH, pmol/L	Cinacalcet dose, mg	Time to target, weeks	Histology	Post-op Ca, mmol/L
2.94	3.04	17.5	30	Asymptomatic	2.61	2.64	Not recorded	30	6	Parathyroid adenoma	2.49
2.87	3.00	30.5	30	No improvement in symptoms	2.87	2.94	29.2	90	Not met	Parathyroid adenoma	2.24
2.77	2.83	17.2	30	Improvement in symptoms	2.56	2.68	18.9	30	5	Parathyroid adenoma	2.29
2.99	3.07	20.9	30	Not noted	2.70	2.78	Not recorded	30	Not met	Parathyroid adenoma	2.15
2.94	3.02	26.9	30	Asymptomatic	2.46	2.47	Not recorded	30	6	Parathyroid adenoma	2.41
3.12	3.22	40.4	30	Not noted	3.20	3.27	73.3	90	Not met	Parathyroid adenoma	2.15
3.06	3.11	19.1	30	Asymptomatic	2.62	2.67	15.9	60	35	Parathyroid adenoma	1.98 (same day discharge)
3.03	3.08	15.4	30	Asymptomatic	2.93	2.86	Not recorded	30	Not met	Parathyroid adenoma	2.58
2.97	3.02	37.9	30	Not noted	2.65	2.71	25.8	60	6	Parathyroid adenoma	2.45
3.09	3.25	25.6	30	Not noted	2.32	2.40	12.3	90	27	Ectopic parathyroid adenoma (two previous parathyroid surgeries)	1.76 (needed IV calcium; 96hr admission)
2.98	3.04	23.0	30	Not noted	2.79	2.85	29.0	60	Not met	Parathyroid adenoma	2.36
2.93	2.98	35.4	30	Not noted	2.45	2.51	24.9	30	6	Parathyroid adenoma/Parathyroid hyperplasia	2.45
2.99	3.05	27.9	30	Not noted	2.73	2.80	9.9	30	Not met	Parathyroid adenoma	2.63
2.91	2.99	19.0	30	Not noted	2.75	2.85	Not recorded	30	Not met	Parathyroid adenoma	2.4
2.93	2.99	14.7	30	Not noted	2.60	2.68	15.8	30	8	Parathyroid adenoma	Not recorded
2.64	2.80	12.1	30	No improvement in symptoms	2.47	2.58	Not recorded	30	4	Parathyroid adenoma	Not recorded
2.86	2.94	16.3	30	Asymptomatic	2.56	2.58	Not recorded	30	6	Parathyroid adenoma	Not recorded
2.79	2.91	18.9	30	Not noted	2.68	2.78	17.5	30	5	Parathyroid adenoma	Not recorded
3.03	3.08	11.3	30	Asymptomatic	3.03	3.06	17.3	30	Not met	Parathyroid adenoma	Not recorded
3.02	3.04	15.2	30	No improvement in symptoms	2.60	2.62	Not recorded	30	18	Parathyroid adenoma	2.45
3.34	3.36	16.9	30	Asymptomatic	2.53	2.58	Not recorded	30	10	Parathyroid adenoma	Not recorded
3.09	3.14	25.8	30	Not noted	2.54	2.65	25.8	60	17	Parathyroid hyperplasia (single gland removed)	2.41
2.92	3.00	8.9	30	Not noted	2.75	2.78	Not recorded	60	17	Parathyroid adenoma	2.45
3.00	2.98	7.3	30	Not noted	2.47	2.43	Not recorded	30	16	Parathyroid adenoma	2.54

Eighteen of these patients had post-operative biochemistry recorded locally within 3 weeks following surgery, mean post-operative Ca 2.40 mmol/L ± 0.14 mmol/L, aCa 2.42 mmol/L ± 0.20 mmol/L and PO 1.22 mmol/L ± 0.25 mmol/L. There were two cases of post-operative hypocalcaemia amongst this subgroup; one patient taking cinacalcet 90 mg/day (highest dose in our cohort) required i.v. calcium and remained an inpatient for 96 h. It should be noted that this patient had a history of two previous parathyroid surgeries and the removal of a single histologically proven normal parathyroid gland during a previous surgery. The third and final surgery for this patient included a hemi-thyroidectomy to remove an intrathyroidal parathyroid adenoma. The second patient that developed post-operative hypocalcaemia was discharged following a targeted surgery for single gland disease on the same day with oral calcium and alfacalcidol.

### Adverse effects attributed to cinacalcet

Sixteen patients reported adverse effects though most were tolerated; only four patients found cinacalcet intolerable and ceased treatment. Nausea and vomiting were the most commonly reported adverse effects in seven patients and caused treatment cessation in three patients. One further patient ceased treatment due to headaches. Hypocalcaemia occurred in four patients though was noted more frequently in patients commenced on 60 mg/day (2 patients out of 8 started on 60 mg/day) vs 30 mg/day (2 patients out of 78 started on 30 mg/day). In all patients, hypocalcaemia resolved with dose reduction permitting the continuation of cinacalcet and all subsequently achieved target Ca. Overall frequency of intolerable adverse effects was similar across low dose cinacalcet ≤ 30 mg/day (3 recorded in 62 patients) and high dose cinacalcet > 30 mg/day (1 in 24).

One patient elected to withdraw all active treatment modalities including cinacalcet for pHPT but reported no adverse effects prior to discontinuation.

### Literature search

Using our search strategy, 278 results were returned, yielding 10 trials and case series reviews which were appraised (Table 3).

**Table 3:** Summary of literature review.

Paper	Study design	Baseline biochemistry	Post-treatment biochemistry	Clinical outcome
		Assay methods (if specified)		
1 (12)	Population: 40 patients in head-to-head then 45 open label; mean age, 64.7. Intervention: cinacalcet titrated to target Ca < 2.58 mmol/L; treated for 235 weeks.	Ca 10.8 mg/dL (2.70 mmol/L) PO 2.65 mg/dL (0.86 mmol/L) PTH 110 pg/mL (11.4 pmol/L)	Ca 9.9 mg/dL (2.48 mmol/L) PO 3.3 mg/dL (1.07 mmol/L) PTH 87.7 pg/mL (9.13 pmol/L)	Final dose: cinacalcet dose 60–120 mg/day. Outcome: 74% achieved target Ca. Adverse effects: in 29% of patients.
		Serum Ca, P, creatinine and AP were measured by standard methods (Covance Central Laboratories, Indianapolis, IN) Intact PTH was measured using a double-antibody IRMA (Allegro PTH; Nichols Institute Diagnostics, San Juan Capistrano, CA)		
2 (13)	Population: 15 patients with MEN1; mean age, 42.3. Intervention: cinacalcet titrated to target aCa < 2.58 mmol/L; treated for 28 weeks.	aCa 11.6 mg/dL (2.88 mmol/L) PTH 97.8 pg/mL (10.19 pmol/L)	aCa 9.5 mg/dL (2.38 mmol/L) PTH 68.5 pg/mL (7.13 pmol/L)	Final dose: cinacalcet mean dose, 48 mg/day. Outcome: 100% achieved target aCa. Adverse effects: in 13% of patients; none stopped cinacalcet.
		Serum intact PTH was measured by a chemiluminescent method (Nichols Institute Diagnostics, San Juan Capistrano, CA, USA), with intra- and interassay coefficients of variation of <4.5 and <10.0%, respectively; all other analytes were determined using standard methods		
3 (14)	Population: 70 patients; mean age, 60. Intervention: cinacalcet titrated pre-PTX; treated for up to 112 weeks.	Ca 11.72 mg/dL (2.93 mmol/L) PTH 149.8 pg/mL (15.6 pmol/L)	Ca 10.2 mg/dL (2.55 mmol/L) PTH 127.7 pg/mL (13.3 pmol/L)	Final dose: cinacalcet dose 60–120 mg/day. Outcome: not specified. Adverse effects: in 42% of patients; 28% stopped cinacalcet.
4 (15)	Population: 135 patients across eight centres; mean age, 63. Intervention: cinacalcet titrated to target Ca (not specified); treated for up to 184 weeks.	aCa 2.85 mmol/L PTH 16.0 pmol/L	aCa 2.47–2.55 mmol/L PTH 12–14 pmol/L	Final dose: cinacalcet dose 15–120 mg/day. Half of the cohort achieved target with 30 mg/day. Outcome: 68% achieved target Ca. Adverse effects: in approximately 20% patients; 4% stopped cinacalcet.
5 (7)	Population: 282 patients across 12 centres including patients pre-PTX; median age, 70. Intervention: cinacalcet titrated to target aCa < 2.60 mmol/L; treated for 52 weeks.	Ca 2.84 mmol/L aCa 2.85 mmol/L PO 0.84 mmol/L PTH 16.2 pmol/L	aCa 2.51 mmol/L PO 0.97 mmol/L PTH 12.5 pmol/L	Final dose: mean cinacalcet dose 51 mg/day. Outcome: 71% achieved target aCa at month 12. Adverse effects: in approximately 20% of patients; 7.6% stopped.
6 (16)	Population: 67 patients; mean age, 69.5. Intervention: cinacalcet titrated to target Ca < 2.58 mmol/L; treated for 28 weeks.	aCa 11.75 mg/dL (2.93 mmol/L) PO 2.66 mg/dL (0.86 mmol/L) PTH 157.5 pg/mL (16.4 pmol/L)	aCa 2.45 mmol/L PO 3.54 mg/dL (1.14 mmol/L) PTH 121.3 pg/mL (12.6 pmol/L)	Final dose: mean cinacalcet dose 82.7 mg/day. Outcome: 75.8% achieved target Ca. Adverse effects: in 42% of patients; 3% stopped cinacalcet.
		All laboratory indices were assayed at three central facilities run by Covance in the USA, Switzerland and Singapore using standardised assay protocols		
7 (8)	Population: 23 patients treated pre-PTX; mean age, 57. Intervention: cinacalcet titrated to target aCa < 2.83 mmol/L; treated for median 9 weeks.	aCa 13.33 mg/dL (3.33 mmol/L) PO 2.7 mg/dL (0.87 mmol/L) PTH 187 pg/mL (19.47 pmol/L)	aCa 10.2 mg/dL (2.55 mmol/L) PTH 134 pg/mL (14.0 pmol/L)	Final dose: median cinacalcet dose, 120 mg/day. Outcome: 83% patients achieved target aCa. Adverse effects: reported in all patients; 4.5% stopped cinacalcet.
		Serum PTH was measured using an electrochemiluminescence (ECLIA) immunoassay (intra-assay coefficient of variation 4.2–6.4%; Cobas 8000, Roche Diagnostics); serum calcium, serum phosphorus, serum albumin and serum creatinine were determined using standard methods.		
8 (17)	Population: 29 patients; mean age, 77. Intervention: cinacalcet titrated to target aCa < 2.60 mmol/L; treated for at least 24 weeks.	aCa 2.80–3.00 mmol/L PO 0.80–0.90 mmol/L PTH 14.8–17.5 pmol/L	aCa 2.25–2.45 mmol/L PTH 10.0–11.1 pmol/L	Final dose: mean cinacalcet dose 68.3–81.8 mg/day. Outcome: 72.4% achieved target aCa. Adverse effects not discussed.
		All biochemical parameters were assayed using Roche Cobas 6000 and 8000 instruments (Roche)		
9 (18)	Population: 61 patients; mean age, 67.8. Intervention: cinacalcet titrated to target aCa < 2.45 mmol/L; treatment period not specified.	aCa 11.0 mg/dL (2.75 mmol/L) PO 2.7 mg/dL (0.87 mmol/L) PTH 181 pg/mL (18.9 pmol/L)	aCa 9.6 mg/dL (2.40 mmol/L) PO 3.2 mg/dL (1.03 mmol/L) PTH 131 pg/mL (13.6 pmol/L)	Final dose: mean cinacalcet dose, 43.4 mg/day. Outcome: 74% achieved target aCa. Baseline aCa significantly higher in patients requiring >50 mg/day. Adverse effects: in 19% of patients; 15% stopped.
		Serum calcium (Ca), albumin, phosphorus and creatinine were measured using LABO‐ SPECT008® (Hitachi, Ltd.) Serum intact PTH was measured using the ECLIA method with a Cobas 6000® (Roche Diagnostics K.K.)		
10 (9)	Population: 110 patients treated pre-PTX; median age, 62. Intervention: cinacalcet titrated to target (not specified); treated for 4 weeks.	Ca 2.60 mmol/L aCa 2.65 mmol/L PO 0.86 mmol/L PTH 10.0 pmol/L	Not specified	Final dose: not specified. Outcome: not specified. Higher baseline PTH, iCa and tumour size predictive of higher dose. Adverse effects: described as frequent GI effects but no patients stopped.

Our cohort is the second largest single-centre cohort and among the oldest reported with a median age of 77 years old (nine out of ten cohorts in the literature reporting a mean age under 70). Baseline biochemistry was similar to our cohorts. A variety of assay methods and reference ranges were utilised in the various studies, accordingly, biochemical targets varied between studies. Our target for titration was among the most lenient at Ca < 2.70 mmol/L; most papers targeted Ca < 2.58 mmol/L. Half of the papers reported a mean dose of cinacalcet below the licensed dose of 60 mg/day, though ours were the lowest reported mean dose. In all papers, mean Ca/aCa normalised and mean PO rose within the normal range. Mean PTH fell in all papers, though normalisation was only seen in a single study (13).

Seven out of the eight papers reporting on the proportion of patients achieving biochemical remission, recording similar outcomes to our study with 68–83% of patients reaching a target Ca or aCa. The remaining paper studying patients with MEN1 was able to achieve target biochemistry in 100% of patients, though this was the smallest reported cohort (13). Two studies (9, 18) noted that higher baseline biochemical parameters, including PTH, correlated with a higher final dose of cinacalcet, in line with our study findings. Thresholds for initiation at a higher dose of cinacalcet were not discussed in the literature.

## Discussion

We have retrospectively analysed a large cohort of patients who received cinacalcet therapy at our centre for the treatment of pHPT. In concordance with published evidence, our data show that cinacalcet is useful at improving the biochemical parameters in pHPT; Ca was normalised in most patients (68 patients) and PTH fell during treatment but remained above the normal range across the cohort. Notably, this large cohort received a lower mean dose of cinacalcet (39.4 mg/day ± 17.1 mg/day) compared to any other published data series, but comparable results were noted in this study in terms of percentage of patients achieving biochemical targets (79.1%). Most patients commenced on low dose cinacalcet of 30 mg/day went on to achieve target Ca without needing to escalate to a full licensed dose of 60 mg/day. Severe biochemical hypercalcaemia necessitates hospitalisation for acute management (19), thus treating hypercalcaemia with cinacalcet avoids the need for hospitalisation. No change in baseline biochemistry was observed prior to the first wave of the pandemic compared to the first 4 months of the pandemic, though as the pandemic progressed and PTX became progressively more delayed, it is possible that more patients would present with higher baseline Ca, aCa or PTH. This would require further study using data from later in the pandemic when surgeries had been delayed for longer periods of time.

Cinacalcet of 30 mg/day was well-tolerated in this study with low rates of treatment cessation caused by adverse effects (4.7% in this study vs 3-28% reported in the literature). Non-adherence due to medication supply or side effects such as nausea and headache were noted among ten patients who failed to achieve the target Ca on 30 mg/day. Utilising a lower dose with less-frequent dosing (i.e. 30 mg once daily, vs 30 mg twice daily) may improve adherence (20), though there are no data specific to cinacalcet that shows improved adherence with lower doses.

Within our cohort, overall adverse effects were not observed more frequently in patients taking doses >30 mg/day. There are few published head-to-head data on the frequency of adverse effects with different doses of cinacalcet; one small study treating patients with secondary hyperparathyroidism observed more adverse effects with a higher dose of cinacalcet (25 mg daily vs 25 mg alternate days), but this did not reach statistical significance (21). Hypocalcaemia was observed more frequently in patients taking higher dose cinacalcet (2 out of 8 patients vs 2 out of 78 patients) though patients initiated at a dose of 60 mg/day in our historical cohort were not stratified according to baseline PTH. In all cases hypocalcaemia resolved with cinacalcet dose reduction, permitting the continuation of treatment. We advocate frequent biochemical monitoring regardless of the initial dose.

Baseline biochemistry was comparable to other published cohorts. We noted significantly higher PTH in patients requiring a dose escalation of cinacalcet above 30 mg/day and performed a ROC curve analysis to determine a PTH threshold for necessitating cinacalcet doses >30 mg/day. We propose a novel PTH threshold of >30 pmol/L for consideration of initiating cinacalcet at 60 mg/day thus aiming to achieve biochemical targets sooner. We would retain a starting dose of 30 mg/day with a PTH value between 25 and 30 pmol/L but employ more frequent biochemical monitoring with a view to earlier dose escalation.

Cinacalcet prior to PTX was endorsed by the European Society for Endocrinology as a bridge during the COVID-19 pandemic in anticipation of reduced access to surgery (10). Twenty-four patients in our cohort were prescribed cinacalcet prior to PTX with a mean final dose of cinacalcet 44.3 mg/day ± 21.4 mg/day. Fifteen patients (62.5%, vs overall 79.1%) achieved a target Ca, eight patients required dose escalation (33.3%, vs overall 20.9%), and the median interval to achieving target Ca was 6 weeks (range, 4–35 weeks). Post-operative hypocalcaemia occurred in two patients receiving cinacalcet, though the only case of post-operative hypocalcaemia prolonging the hospital admission was noted in a complex symptomatic patient taking a high dose of cinacalcet 90 mg/day prior to a third parathyroid surgery. No significant difference in mean biochemistry was identified between the two groups, although it should be noted that vitamin D levels were not routinely recorded and therefore not analysed for these cohorts. These data support the utility of using cinacalcet as a bridge to surgery, and this is an important consideration as we anticipate ongoing delays locally and nationally for elective PTX due to COVID-19. At our centre, waiting lists for parathyroid imaging and elective PTX have lengthened beyond pre-pandemic levels, which have resulted in more frequent usage of cinacalcet as a bridge to curative surgical treatment to avoid hospitalisations with hypercalcaemia and irreversible end-organ damage. The local frequency of cinacalcet initiation approximately doubled during the first 3 months of the pandemic from March 2020 to July 2020 (3.6 patients/month) compared to the period of January 2017 to February 2020 (1.76 patients/month).

Compared to using full licensed dose cinacalcet of 60 mg/day, lower dose cinacalcet therapy also grants additional drug acquisition cost savings; 50 patients achieved target Ca on doses under the full dose of 60 mg/day and received therapy for a mean duration of 12 months, generating a cohort-wide saving of £64,486.32 compared to 50 patients receiving the full dose of 60 mg/day (drug prices taken from the September 2020 online Drug Tariff Part VIIIA and are exclusive of VAT).

### Limitations

There were limitations to our analysis largely related to the retrospective design. A standardised pro forma was not used in the assessment of pre- or intra-treatment symptom improvement on cinacalcet in the pHPT clinic, and changes in symptoms on treatment were not consistently recorded, as such we cannot draw conclusions as to whether cinacalcet improves symptoms of hypercalcaemia, either for long-term medical treatment or for bridging prior to PTX. We were unable to analyse other sequelae of pHPT including changes in bone mineral density and nephrolithiasis as these were not consistently recorded across the cohort; although it should be noted that current evidence does not consistently demonstrate improvement in these aspects with cinacalcet. Vitamin D was neither consistently tested prior to treatment nor was it recorded whether patients were taking supplementary vitamin D prior to or during treatment with cinacalcet. This was a non-controlled analysis, which further limits the scope of conclusions that can be drawn from these data.

## Conclusion

In summary, we report that low dose cinacalcet (30 mg/day) is useful at treating hypercalcaemia driven by pHPT in patients with symptomatic or severe hypercalcaemia, including patients awaiting PTX. No conclusions can be drawn from these data as to whether cinacalcet improves symptoms of hypercalcaemia. Low dose cinacalcet (30 mg daily) was generally well-tolerated and represents an opportunity for drug acquisition cost saving compared to full licensed dose cinacalcet (60 mg/day). Post-operative hypocalcaemia resulting in prolonged hospital admissions may be more common with higher doses of cinacalcet or in those patients having repeat parathyroid surgeries and close post-operative surveillance for hypocalcaemia should be considered in such patients. We have identified a high baseline PTH as a risk factor for necessitating further titration of cinacalcet and proposed a baseline PTH > 30 pmol/L for initiating at full dose of 60 mg/day, though patients given 60 mg/day are at greater risk of hypocalcaemia. In practice, we intend to utilise an initial cinacalcet dose of 30 mg/day in most patients, except those with baseline PTH > 30 pmol/L, with a view to reduce drug acquisition costs and possibly reducing the incidence of hypocalcaemia, without diminishing the effect on Ca.

## Supplementary Material

Supplementary Materials

## Declaration of interest

B G C is an employee of AstraZeneca. The other authors have nothing to disclose.

## Funding

This work did not receive any specific grant from any funding agency in the public, commercial or not-for-profit sector.

## References

[1] Melton 3rd LJ Epidemiology of primary hyperparathyroidism. Journal of Bone and Mineral Research 1991 6 (Supplement 2) S25–S30; discussion S1–S2. (10.1002/jbmr.5650061409) 1763669

[2] Bilezikian JP Brandi ML Eastell R Silverberg SJ Udelsman R Marcocci C Potts Jr JT. Guidelines for the management of asymptomatic primary hyperparathyroidism: summary statement from the Fourth International Workshop. Journal of Clinical Endocrinology and Metabolism 2014 99 3561–3569. (10.1210/jc.2014-1413) 25162665 PMC5393490

[3] NHS England Specialised Services Clinical Reference Group for Specialised Endocrinology. Clinical Commissioning Policy: Cinacalcet for complex primary hyperparathyroidism in adults. 16034/P, July 2016.

[4] Leere JS Karmisholt J Robaczyk M Vestergaard P. Contemporary medical management of primary hyperparathyroidism: a systematic review. Frontiers in Endocrinology 2017 8 79. (10.3389/fendo.2017.00079) PMC5397399 28473803

[5] Cetani F Saponaro F Marcocci C. Non-surgical management of primary hyperparathyroidism. Best Practice and Research: Clinical Endocrinology and Metabolism 2018 32 821–835. (10.1016/j.beem.2018.09.006) 30665549

[6] NHS England Specialised Services Clinical Reference Group for Specialised Endocrinology. Clinical Commissioning Policy: Cinacalcet for complex primary hyperparathyroidism in adults (16034/P). Revised: Feb 2017. (available at: https://www.england.nhs.uk/wp-content/uploads/2017/06/ccp-cinacalcet-complex-primary-hyperparathyroidism-adults.pdf)

[7] Schwarz P Body JJ Cáp J Hofbauer LC Farouk M Gessl A Kuhn JM Marcocci C Mattin C Muñoz Torres M***et al***. The PRIMARA study: a prospective, descriptive, observational study to review Cinacalcet use in patients with primary hyperparathyroidism in clinical practice. European Journal of Endocrinology 2014 171 727–735. (10.1530/EJE-14-0355) 25240499

[8] Misiorowski W Zgliczyński W. Cinacalcet as symptomatic treatment of hypercalcaemia in primary hyperparathyroidism prior to surgery. Endokrynologia Polska 2017 68 306–310. (10.5603/EP.2017.0023) 28660989

[9] Koman A Ohlsson S Bränström R Pernow Y Bränström R Nilsson IL. Short-term medical treatment of hypercalcaemia in primary hyperparathyroidism predicts symptomatic response after parathyroidectomy. British Journal of Surgery 2019 106 1810–1818. (10.1002/bjs.11319) 31595982

[10] Gittoes NJ Criseno S Appelman-Dijkstra NM Bollerslev J Canalis E Rejnmark L Hassan-Smith Z. Endocrinology in the time of COVID-19: management of calcium disorders and osteoporosis. European Journal of Endocrinology 2020 183 G57–G65. (10.1530/EJE-20-0385) 32396134 PMC7938011

[11] Casey R Bell D. Cambridgeshire and Peterborough Clinical Commissioning Group Shared Care Guideline: cinacalcet for the management of PRIMARY hyperparathyroidism in patients with severe hypercalcaemia awaiting surgery or deemed unfit for surgical management. Version 1 2020. (available at: https://www.cambridgeshireandpeterboroughccg.nhs.uk/easysiteweb/getresource.axd?assetid=21548)

[12] Peacock M Bolognese MA Borofsky M Scumpia S Sterling LR Cheng S Shoback D. Cinacalcet treatment of primary hyperparathyroidism: biochemical and bone densitometric outcomes in a five-year study. Journal of Clinical Endocrinology and Metabolism 2009 94 4860–4867. (10.1210/jc.2009-1472) 19837909

[13] Filopanti M Verga U Ermetici F Olgiati L Eller-Vainicher C Corbetta S Persani L Beck-Peccoz P Spada A. MEN1-related hyperparathyroidism: response to Cinacalcet and its relationship with the calcium-sensing receptor gene variant Arg990Gly. European Journal of Endocrinology 2012 167 157–164. (10.1530/EJE-12-0117) 22577108

[14] Norman J Lopez J Politz D. Cinacalcet (Sensipar) provides no measurable clinical benefits for patients with primary hyperparathyroidism and may accelerate bone loss with prolonged use. Annals of Surgical Oncology 2012 19 1466–1471. (10.1245/s10434-011-2065-9) 21922336

[15] Saponaro F Faggiano A Grimaldi F Borretta G Brandi ML Minisola S Frasoldati A Papini E Scillitani A Banti C***et al***. Cinacalcet in the management of primary hyperparathyroidism: post marketing experience of an Italian multicentre group. Clinical Endocrinology 2013 79 20–26. (10.1111/cen.12108) 23228121

[16] Khan A Bilezikian J Bone H Gurevich A Lakatos P Misiorowski W Rozhinskaya L Trotman ML Tóth M. Cinacalcet normalizes serum calcium in a double-blind randomized, placebo-controlled study in patients with primary hyperparathyroidism with contraindications to surgery. European Journal of Endocrinology 2015 172 527–535. (10.1530/EJE-14-0877) 25637076 PMC5729741

[17] Abusahmin H Surya A Aldridge A Okosieme O Das G. Cinacalcet: a viable therapeutic option for primary hyperparathyroidism in the elderly. Indian Journal of Endocrinology and Metabolism 2018 22 485–488. (10.4103/ijem.IJEM_684_17) 30148094 PMC6085958

[18] Manaka K Sato J Kinoshita Y Ito N Fujita M Iiri T Nangaku M Makita N. Effectiveness and safety of Cinacalcet for primary hyperparathyroidism: a single center experience. Endocrine Journal 2019 66 683–689. (10.1507/endocrj.EJ19-0034) 31092749

[19] Walsh J Gittoes N Selby P & Society for Endocrinology Clinical Committee. SOCIETY FOR ENDOCRINOLOGY ENDOCRINE EMERGENCY GUIDANCE: Emergency management of acute hypercalcaemia in adult patients. Endocrine Connections 2016 5 G9–G11. (10.1530/EC-16-0055) 27935816 PMC5314807

[20] Jin J Sklar GE Min Sen Oh V Chuen Li S. Factors affecting therapeutic compliance: a review from the patient’s perspective. Therapeutics and Clinical Risk Management 2008 4 269–286. (10.2147/tcrm.s1458) 18728716 PMC2503662

[21] Gojaseni P Pattarathitinan D Chittinandana A. Efficacy of low-dose Cinacalcet on alternate days for the treatment of secondary hyperparathyroidism in hemodialysis patients: a single-center study. International Journal of Nephrology and Renovascular Disease 2017 10 47–53. (10.2147/IJNRD.S124844) 28223837 PMC5304993

