# Supplementary material for: A single-centre retrospective analysis of cinacalcet therapy in primary hyperparathyroidism
Source: Endocr Connect. 2021 Oct 14;10(11):1435–44. doi: 10.1530/EC-21-0258 (PMC8630765; doi:10.1530/EC-21-0258)
Supplement: Supplementary Materials [file supplementary_material.pdf]

Supplementary Appendix

Cinacalcet prescribing pathway following local Share Care Guideline [11]

Cinacalcet will be funded by local commissioners for the treatment of pHPT in patients who are not deemed suitable for parathyroid surgery with serum calcium:

a) >2.9mmol/L with symptoms related to hypercalcaemia
b) >3.0mmol/L regardless of symptoms

↓

Cinacalcet will be commenced by the endocrinologists in the pHPT clinic at a dose of 30mg, taken orally, once daily.

↓

If calcium has not fallen to <2.7mmol/L, or by a minimum of 0.25mmol/L, in the first eight weeks the dose of cinacalcet should be increased by endocrinologists in the pHPT clinic to 30mg, taken orally, twice daily.

↓

The dose should be further adjusted by endocrinologists in the pHPT clinic to achieve target calcium <2.7mmol/L.

↓

Once calcium is in the target range on a stable dose of cinacalcet prescribing responsibility will transfer to the general practitioner.

↓

The GP should monitor calcium every three months and seek advice from the endocrinologists in the pHPT clinic if calcium falls <2.4mmol/L or rises >2.7mmol/L.
